# Inhibition of ferric salts on phosphorus-accumulating organisms in simultaneous chemical precipitation for phosphorus removal

**DOI:** 10.3389/fmicb.2025.1681450

**Published:** 2025-10-28

**Authors:** Anqi Xiao, Jianbo Yu, Ziyuan Lin, Meng Cao, Shuai Jian, Shuxuan Lin, Jian Zhou

**Affiliations:** ^1^Key Laboratory of the Three Gorges Reservoir Region’s Eco-Environment, Ministry of Education, Chongqing University, Chongqing, China; ^2^Chongqing Water & Environment Holdings Group, Ltd., Chongqing, China

**Keywords:** biological phosphorus removal, chemical phosphorus removal, ferric salts, phosphorus fraction, phosphate-accumulating organisms

## Abstract

To comply with the increasingly strict phosphorus (P) effluent standard, ferric salts are commonly used as a simultaneous precipitant to supplement the biological P removal process. However, ferric residue from the chemical process can be carried by the return sludge into the biological system, potentially affecting the biological P removal process. This study demonstrated that ferric salts had significant inhibitory effects on the biological P removal process. The activity and relative abundance of phosphate-accumulating organisms (PAOs) decreased after adding ferric salts. P uptake rate dropped from 10.31 to 2.39 mg/(g SS⋅h) and the relative abundance of PAOs decreased from 2.51% to 0.21% when ferric salts increased from 0 to 42 mg Fe^3+^/L. P release and uptake by PAOs were inhibited, and bioavailable P and poly-P in the sludge dropped after ferric addition. As a result, the chemical P removal with ferric precipitation contributed more to P removal, and the ortho-P in the sludge spiked. The inhibition of the biological P removal process may make it difficult to control simultaneous ferric dosing, where the biological P removal process is required to make a major contribution to P removal.

## 1 Introduction

The discharge of phosphorus-rich wastewater can lead to eutrophication of water bodies (Abdoli et al., 2024). The enhanced biological phosphorus removal (EBPR) process has been widely applied to remove phosphorus (P) from various phosphorus-rich wastewater (Di Capua et al., 2022; Diaz et al., 2022). The EBPR process can achieve the effluent P concentration of 0.5∼1 mg/L. However, regulations on P discharge limits have become more stringent to prevent eutrophication. The total phosphorus (TP) in effluent stipulated by the Discharge Standard of Pollutants for Municipal Wastewater Treatment Plants (GB 18918–2002) in China is less than 0.5 mg/L. To meet the TP effluent standard, EBPR has been supplemented with chemical P removal, where ferric chloride is commonly used as a simultaneous precipitant in the activated sludge system (Caravelli et al., 2010; Zhang et al., 2015). In the simultaneous chemical P removal system, ferric chloride is usually added at the end of the aerobic zone. It precipitates with the residual P in the effluent of the EBPR process to further reduce the P level to meet the standard. However, the ferric chloride could flow back into the anaerobic zone along with the sludge reflux, which may affect the EBPR process.

The EBPR process relies on phosphorus-accumulating organisms (PAOs) that anaerobically release P and aerobically take up excess P inside the cells (Seviour et al., 2003). P is then removed from wastewater by wasting phosphorus-rich sludge. Ferric salts that flow back into the anaerobic zone through the sludge reflux may rapidly undergo co-precipitation with P in the influent, which can increase the C/P ratio by decreasing the available P content in wastewater. The C/P ratio can influence microbial communities. A high C/P ratio is more conducive to the proliferation of glycogen-accumulating organisms (GAOs) (Burow et al., 2008). On the other hand, under high C/P, PAOs could also shift their metabolism from polyphosphate accumulation to glycogen accumulation, and biological P removal was compromised (Acevedo et al., 2017). Additionally, the metabolic activity of PAOs is sensitive to various metal ions (Chen et al., 2019; Sheng et al., 2025). Micro-dosing of ferrous was found to enhance the EBPR process (Ji et al., 2020), while a large amount of ferrous ions had inhibitory effects on PAOs activity (Xia et al., 2022). Similarly, high levels of aluminum salts inhibited the biological P removal and nitrification processes by suppressing the activity of activated sludge (Liu et al., 2011). The activity of PAOs may be restricted either by ferric-induced changes in the C/P ratio or by metal toxicity. Most studies have focused on the impact of metal coagulants on the performance of biological treatment systems regarding chemical oxygen demand (COD) and P removal, and the physicochemical properties of sludge. There has been little research on the influence of ferric iron on the metabolic pathways and microbial communities of the biological P removal system.

This study aims to investigate the influence of ferric on the EBPR process. The study was carried out in a sequencing batch reactor (SBR) operating under alternating anaerobic/aerobic conditions. Firstly, the long-term effect of different ferric concentrations on the P removal performance of the EBPR process was explored. Then, the different forms of P in sludge were analyzed to identify the change in P removal pathways with ferric dosing. Finally, the effects of ferric dosing on microbial community structure were identified.

## 2 Materials and methods

### 2.1 Experimental set-up and operation

The SBR was a cylindrical vessel made of Plexiglas with an effective volume of 2 L. The inoculated sludge in the start-up stage of the reactor was obtained from a wastewater treatment plant (Chongqing, China). The mixed liquor volatile suspended solids (MLVSS) were 5 g/L. A magnetic mixer was placed at the bottom of the reactor to maintain the activated sludge suspended during the reaction. Oxygen was supplied through an aerator. The gas flow rate was measured and controlled by a gas rotameter. Dissolved oxygen (DO) was kept at 2.0∼3.0 mg/L during the aerobic period. A time-controlled switch was used to manage the batch operation of SBR with a drainage ratio of 0.5. The operation cycle was 8 h, consisting of feeding (instant), anaerobic phase (3 h), aerobic phase (4.5 h), settling (0.5 h), and decanting (instant). The external heating jacket was utilized to maintain the reactor temperature at 20 °C ± 1 °C. SBR was operated under different ferric ion concentrations (14 ± 2, 28 ± 2, 42 ± 2 mg/L) by adding ferric chloride to investigate the effects of ferric salts on the biological P removal process.

### 2.2 Characteristics of wastewater

The synthetic wastewater was prepared based on municipal wastewater. Sodium acetate/sodium propionate, potassium dihydrogen phosphate, and ammonium chloride were used as key sources of organic matter, P, and N, respectively. COD, PO_4_^3–^-P and NH_4_^+^-N were 400 ± 50, 20 ± 2, 10 ± 2 mg/L, respectively. The influent pH was maintained at 7.3 ± 0.2 by adding potassium bicarbonate. Trace element stock solution was used to ensure the supply of nutrients required for microbial growth, with its formula shown in Table 1. 1 ml trace element stock solution was added to 1 L of synthetic wastewater.

**TABLE 1:** Composition of trace element solution.

Chemical compound	Concentration (g/L)
FeCl_3_⋅6H_2_O	1.50
H_3_BO_3_	0.15
CuSO_4_⋅5H_2_O	0.03
KI	0.18
MnCl_2_⋅4H_2_O	0.12
Na_2_MoO⋅2H_2_O	0.06
ZnSO_4_⋅7H_2_O	0.12
COCl_2_⋅6H_2_O	0.15
EDTA	5.00

### 2.3 Wastewater sampling and analysis

Daily wastewater samples were obtained from the influent and effluent, and at the end of the anaerobic phase throughout the experiment, with hourly samples taken during the typical cycle tests. COD, PO_4_^3–^-P, NH_4_^+^-N, iron ions (Fe^2+^ and Fe^3+^), SS, and TSS were measured according to the standard methods (APHA, 2012). pH and DO were determined by a multi-parameter analytical instrument (Hach HQ30d, USA).

### 2.4 Morphology and element composition of sludge

The formation of FePO_4_ precipitates after ferric addition may change the morphology and element composition of the activated sludge. The sludge samples collected from SBR were cleaned and dried in an oven at 105 °C for 12 h and then ground into powder for the test. Scanning electron microscopy images combined with energy dispersive spectrometry (SEM-EDS, ZEISS EV018, Germany) were collected for the sludge surface morphology observation and chemical elements composition screening.

### 2.5 Determination of phosphorus fractions in sludge and EPS

The phosphorus fractions in sludge were determined based on the Standard Measurements and Testing (SMT) of the European Standards Testing and Measurement Organization (Loh et al., 2020). The sludge samples were first freeze-dried, and then an equal weight of the dried samples was taken for the experiment. Total phosphorus (TP), inorganic phosphorus (IP), organic phosphorus (OP), apatite inorganic phosphorus (AP, the P fraction associated with Ca), and non-apatite inorganic phosphorus (NAIP, the P fraction associated with oxides and hydroxides of Al, Fe, and Mn) were determined using the SMT method.

Extracellular polymeric substance (EPS) was extracted by the ultrasonic-centrifugation method (Li et al., 2019). The TP content in EPS was measured according to the molybdenum blue method in standard methods (APHA, 2012). Different forms of inorganic phosphorus and organophosphorus in sludge were analyzed by a ^31^P nuclear magnetic resonance (^31^P NMR, Agilent Pro Pulse 500, USA) spectroscopy.

### 2.6 16S rRNA high-throughput sequencing analysis

Three sludge samples, each in triplicate, were obtained at the end of different ferric dosing phases (0, 14, 42 mg/L) from the SBR for microbial community analysis. Genomic DNA was extracted from biomass using the PowerSoil DNA isolation kit (MoBio Laboratories) following the manufacturer’s instructions. The extracted DNA was amplified with primers. Primers 338F(ACTCCTACGGGAGGCAGCAG) and 806R(GGACTACHVGGGTWTCTAAT) were used to target the highly altered V3-V4 region for 16S rRNA gene amplification. The 16S rRNA gene was sequenced on the Illumina MiSeq platform. The specific process of amplification was first 95 °C for 5 min followed by 35 thermal cycles at intervals of 30 s, then 55 °C for 30 s and 72 °C for 1 min, and finally 72 °C for 10 min. Amplification was verified in a 2.0% (wt/v) agarose gel electrophoresis fragment using the QuantiFluor™-ST system (Promega, USA). After successful amplification, the PCR products were sent to BGI Genomics (Wuhan, China) for sequencing.

## 3 Results and discussion

### 3.1 Phosphorus removal performance under different ferric dosages

**FIGURE 1:**
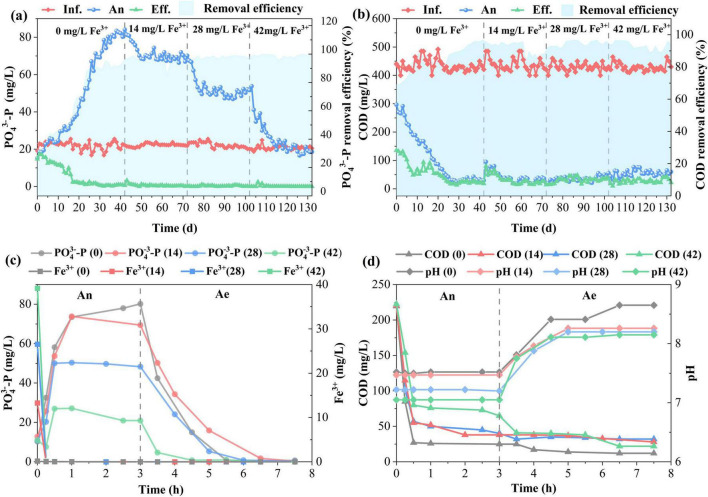
Phosphorus removal performance under different Fe^3+^ concentrations; Daily variation of PO_4_^3–^-P **(a)** and COD **(b)** in the influent (Inf.), end of anaerobic phase (An), effluent (Eff.); The cyclic profiles of PO_4_^3–^-P, Fe^3+^ **(c)**, COD, and pH **(d)** in the typical operation cycle. Ae, aerobic phase.

Figure 1 shows the effects of Fe^3+^ dosing on P removal during long-term operation. After a rapid start-up, SBR gradually stabilized and maintained excellent P removal performance (Figure 1a). A significant decrease in effluent P concentration was observed with an increase in Fe^3+^ dosing (one-way ANOVA, *p* < 0.05). Average effluent P concentration was decreased from 0.66 ± 0.25 (0 mg/L Fe^3+^), 0.49 ± 0.15 (14 mg/L Fe^3+^), 0.29 ± 0.25 (28 mg/L Fe^3+^) to 0.06 ± 0.05 mg/L (42 mg/L Fe^3+^) with corresponding P removal efficiency rising from 97.0%, 98.1%, 98.6% to 99.8%. Fe^3+^ dosing supplemented the biological P removal by chemical precipitation of phosphate, where Fe^3+^ combined with PO_4_^3–^ to form stable FePO_4_ precipitates (Chen et al., 2024). Therefore, P removal efficiency improved. However, Fe^3+^ dosing had apparent inhibitory effects on P release during the anaerobic (An) phase. The amount of P at the end of the anaerobic phase dropped from 80.42 ± 2.91 (0 mg/L Fe^3+^) to 20.24 ± 2.42 mg/L (42 mg/L Fe^3+^). In theory, the maximum P removed by 1 mg of ferric iron (Fe^3+^) is 0.55 mg by assuming all ferric ions react with phosphate to form FePO_4_. 23.1 mg/L P could be removed by ferric precipitation when the ferric concentration was 42 mg/L. Considering chemical P removal, total P released by PAOs was 40.48 mg/L under 42 mg/L Fe^3+^, which was much less than the control group (80.42 ± 2.91 mg/L) without ferric addition. As shown in Figure 1b, COD at the end of the anerobic phase increased with an increase in ferric dosage, which spiked from 33.18 ± 4.54 (0 mg/L Fe^3+^) to 57.89 ± 8.85 mg/L (42 mg/L Fe^3+^). Influent COD was consumed by PAOs as carbon to drive P release during the anaerobic phase. The inhibition of P release by ferric led to an increase in COD. Based on the metabolic model of PAOs, the typical ratio of mg P_*release*_/mg COD_*uptake*_ is 0.5 (Oehmen et al., 2007). 140 and 81 mg/L COD were consumed by PAOs during the anaerobic phase under 0 and 42 mg/L Fe^3+^, respectively. The rest of the COD was degraded by other heterotrophic bacteria. There was no significant difference (one-way ANOVA, *p* > 0.05) in effluent COD under different ferric concentrations. The effluent COD was around 25 mg/L with a removal efficiency of 95%.

The biological P removal was achieved by sequential P release and uptake by PAOs under alternating anaerobic/aerobic (Ae) conditions. As shown in Figure 1c, the dosed Fe^3+^ rapidly dropped to zero within the initial 15 min of the anaerobic phase, indicating a rapid initial P removal upon the addition of ferric salts. Fe^3+^ precipitated with phosphate to form FePO_4_. The maximum P precipitated by ferric was around 0 (0 mg/L Fe^3+^), 7.7 (14 mg/L Fe^3+^), 15.4 (28 mg/L Fe^3+^), and 23.1 mg/L (42 mg/L Fe^3+^), respectively. The chemical P precipitation at the initial 15 min resulted in an increase in the C/P ratio from 3.13 (0 mg/L Fe^3+^) to 20.37 (42 mg/L Fe^3+^). High C/P ratios did not favor PAO metabolism (Schuler and Jenkins, 2003). The amount of P available for PAOs to take up and store as poly-P was significantly reduced after the addition of ferric ions (one-way ANOVA, *p* < 0.05). PAOs can behave metabolically like GAOs as a survival strategy, showing low ratios of P_*release*_/COD_*uptake*_ when intracellular poly-P content is reduced (Acevedo et al., 2012). Thus, the amount of P released dramatically decreased after ferric dosing. P concentration at the end of the anaerobic phase was 80.24 (0 mg/L Fe^3+^), 69.44 (14 mg/L Fe^3+^), 45.12 (28 mg/L Fe^3+^), and 20.93 (42 mg/L Fe^3+^) mg/L, respectively. Considering P removal by chemical precipitation, the total P released by PAOs was 80.24 (0 mg/L Fe^3+^), 77.14 (14 mg/L Fe^3+^), 60.52 (28 mg/L Fe^3+^), and 44.03 (42 mg/L Fe^3+^) mg/L, respectively. PAOs quickly depleted their internal poly-P stores to generate energy for COD uptake, mostly happening in the first 30 min (Figure 1d). As poly-P formation was reduced after ferric addition, the COD uptake rate decreased from 77.01 ± 5.7 (0 mg/L Fe^3+^) to 50.87 ± 3.2 (42 mg/L Fe^3+^) mg COD/(g SS⋅h) at the initial 30 min. This resulted in a high COD (65 mg/L) at the end of the anaerobic phase under 42 mg/L Fe^3+^, which continued to decrease during the aerobic phase. The COD (25 mg/L) was rather low without ferric addition at the end of the anaerobic phase, and it could not further decrease during the aerobic phase. The decrease in P release and COD uptake at the anaerobic phase after the addition of ferric salts suggested that PAOs metabolism was inhibited by ferric salts. The chemical P removal with the addition of ferric salts reduced the available P for biological P uptake during the aerobic phase. The P uptake rate significantly decreased after ferric dosing (one-way ANOVA, *p* < 0.05). The P uptake rate was 10.31 ± 1.03, 5.17 ± 0.86, 4.56 ± 0.67, and 2.39 ± 0.52 mg/(g SS⋅h) when ferric was 0, 14, 28, and 42 mg/L, respectively. A one-way ANOVA was used to test if P uptake rates differed across four ferric ion concentrations (0, 14, 28, and 42 mg/L). The analysis compared the variance between these groups to the variance within them (in triple). The resulting *p*-value was less than 0.05, leading to the rejection of the null hypothesis. This confirmed that the observed decrease in mean P uptake with increasing ferric dose was statistically significant and not due to random chance. The result suggested that the biological P removal was inhibited in the presence of ferric, and biological P removal by PAOs was substantially reduced. Even though ferric salts have inhibitory effects on PAOs, the P in effluents after ferric addition is lower compared to the control without ferric dosing.

### 3.2 Change of phosphorus removal pathway by ferric addition

**FIGURE 2:**
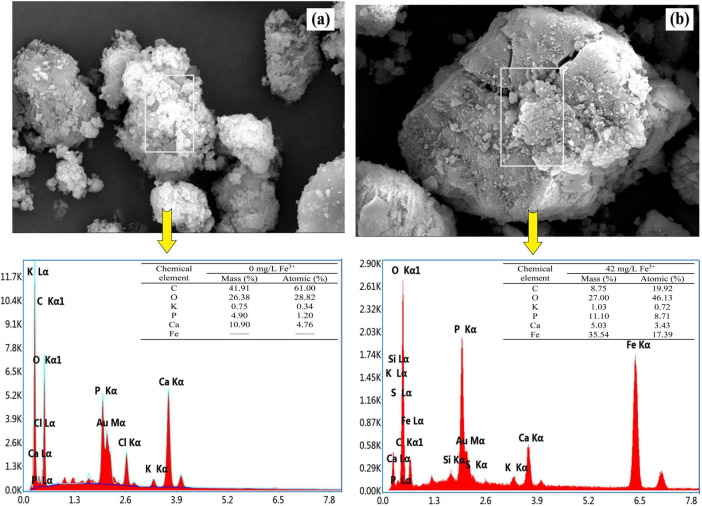
Scanning electron microscopy (SEM) micrograph and EDS of the sludge taken at the end of the anaerobic phase without **(a)** and with (42 mg/L Fe^3+^) **(b)** ferric dosing.

Morphology and composition of the activated sludge are shown in Figure 2. SEM images clearly showed that inorganic sediments were attached to the sludge surface. The surface of the activated sludge without ferric addition was rougher and looser compared to that with ferric addition, indicating that bio-flocculation was enhanced under Fe^3+^ concentration of 44 mg/L for a long time. The EDS analysis revealed sharp peaks for typical elemental compositions of municipal activated sludge, including phosphorus, magnesium, potassium, and calcium (Yu et al., 2022). In addition to these ions, the Fe peak was found in the activated sludge with ferric addition, suggesting that Fe-containing compounds entered the biomass (Figure 2b). This was attributed to chemical precipitation of phosphates by ferric salts. Compounds such as FePO_4_(s) and Fe(OH)_3_(s) have been considered to be precipitated when pH values were between 5 and 8 (Figure 1d; Caravelli et al., 2010). These precipitates were then entrapped in the sludge matrix. Sludge with ferric addition exhibited a lower carbon (C) content but a higher phosphorus (P) content compared to sludge without ferric addition. This was due to the deposition of inorganic FePO_4_(s) and Fe(OH)_3_(s) in sludge.

**FIGURE 3:**
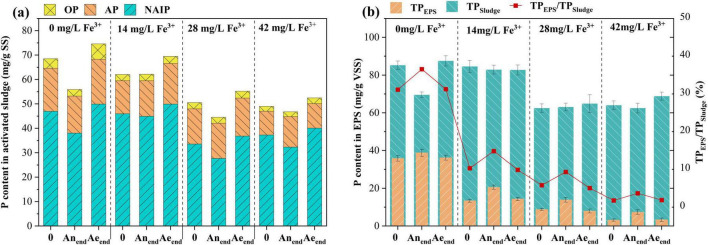
Phosphorus fractions and distributions of the sludge **(a)** and EPS **(b)** under different ferric dosages.

The various forms of P in the sludge and EPS under different ferric dosages were detected according to the SMT (Figure 3). IP (IP = NAIP + AP) was the major P form in all sludge samples (82.5%∼88.2% of TP) (Figure 3a). Compared with IP, OP content was relatively lower, about 3.5%∼7.2% of TP. NAIP and OP are collectively referred to as releasable and bioavailable P (Yu et al., 2024; Zhang et al., 2025). P content in NAIP form at the end of the aerobic phase (Ae_*end*_) decreased when ferric concentration was over 14 mg/L. P content in OP form at the end of the aerobic phase decreased once ferric salt was added. Additionally, the changes in NAIP and OP form from the beginning (0) to the end of the anaerobic phase (An_*end*_), from the beginning (An_*end*_) to the end of the aerobic phase (Ae_*end*_), also dropped with the increase in ferric concentration. EPS serves as a primary P reservoir in flocculent sludge, facilitating biological P removal through the retention of ortho-, pyro-, and poly-phosphate (Li et al., 2015). As shown in Figure 3b, the TP content in EPS decreased considerably with the addition of ferric. The lower NAIP, OP, and EPS-P contents in the sludge with ferric addition demonstrated that the activity of PAOs was inhibited, and biological accumulation and adsorption for P removal were reduced.

**FIGURE 4:**
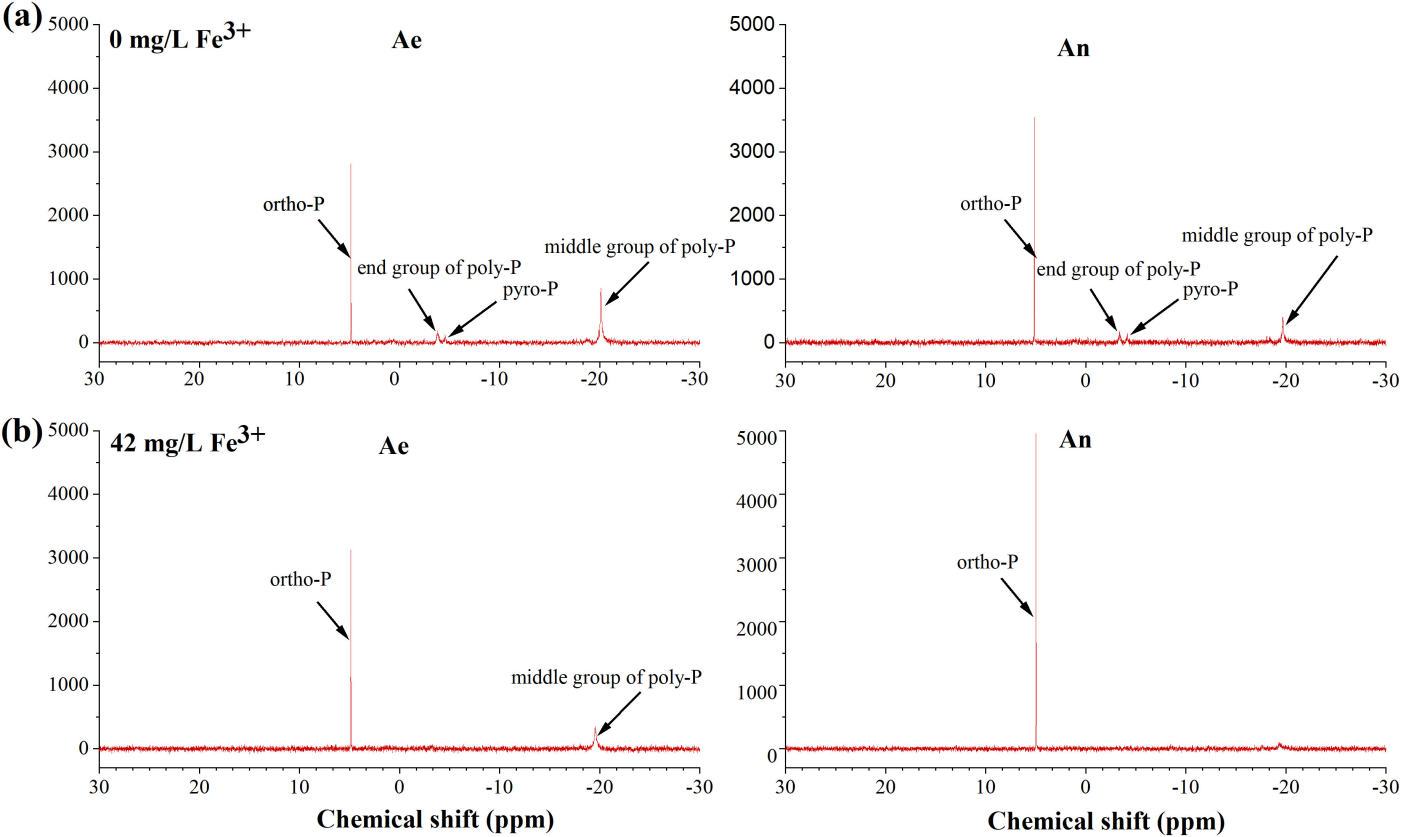
^31^P nuclear magnetic resonance (^31^P NMR) spectra of the sludge taken at the end of the anaerobic phase and aerobic phase without **(a)** and with (42 mg/L Fe^3+^) **(b)** ferric dosing.

^31^P nuclear magnetic resonance spectra show peaks in the areas for orthophosphate (ortho-P, 6 to 7 ppm), pyrophosphate (pyro-P, −4 to −6 ppm), and polyphosphate (end poly-P, −3 to −4 ppm; middle poly-P, −17 to −19 ppm) (Figure 4). The poly-P middle and end groups, ortho-P, and pyro-P were identified in the sludge without Fe^3+^ dosing, while only the poly-P middle group and ortho-P were identified in the sludge with Fe^3+^ dosing. Ortho-P mainly exists in phosphate form, such as Fe- and Ca-bound inorganic P. Ortho-P increased in the anaerobic phase due to the Ca^2+^ and Fe^3+^ in the influent precipitating with phosphate. Thus, the amount of ortho-P was greater in the sludge with the addition of ferric than that without the addition of ferric. Poly-P was the main P species generated by PAOs, and its content in anaerobic sludge decreased due to P release by PAOs. However, the poly-P mass and change were lower in the activated sludge with ferric dosing. NAIP mainly comprises poly-P, orth-P, and pyro-P (Ni et al., 2020). It could be seen that NAIP was mainly metabolized through changes in poly-P in the cells of PAOs, which had higher biological P metabolism than that with ferric dosing. After ferric addition, NAIP was mainly metabolized in the form of ortho-P in the sludge. Therefore, biological P metabolism was inhibited, and some of the P was removed by chemical precipitation after the addition of ferric salts.

### 3.3 Microbial community shifts

The diversity of microbial communities under different Fe^3+^ concentrations is shown in Table 2. The sequencing coverage rates of all sludge samples exceeded 99.5%, indicating that the given data was sufficient to cover all species and had statistical significance. After the addition of Fe^3+^, the Chao index and ACE index dropped, revealing that the richness of the microbial community decreased. The Shannon index decreased, and the Simpson index increased after ferric addition. This indicated that some microorganisms might not be able to adapt to low concentrations of P, and the diversity of the microbial community decreased.

**TABLE 2:** Richness and diversity of microbial communities under different Fe^3+^ concentrations.

Sample	ACE	Chao	Shannon	Simpson	Coverage
0 mg Fe^3+^/L	1109	1114	5.07	0.0236	0.996
14 mg Fe^3+^/L	910	884	4.64	0.0230	0.996
42 mg Fe^3+^/L	933	950	4.23	0.0479	0.996

**FIGURE 5:**
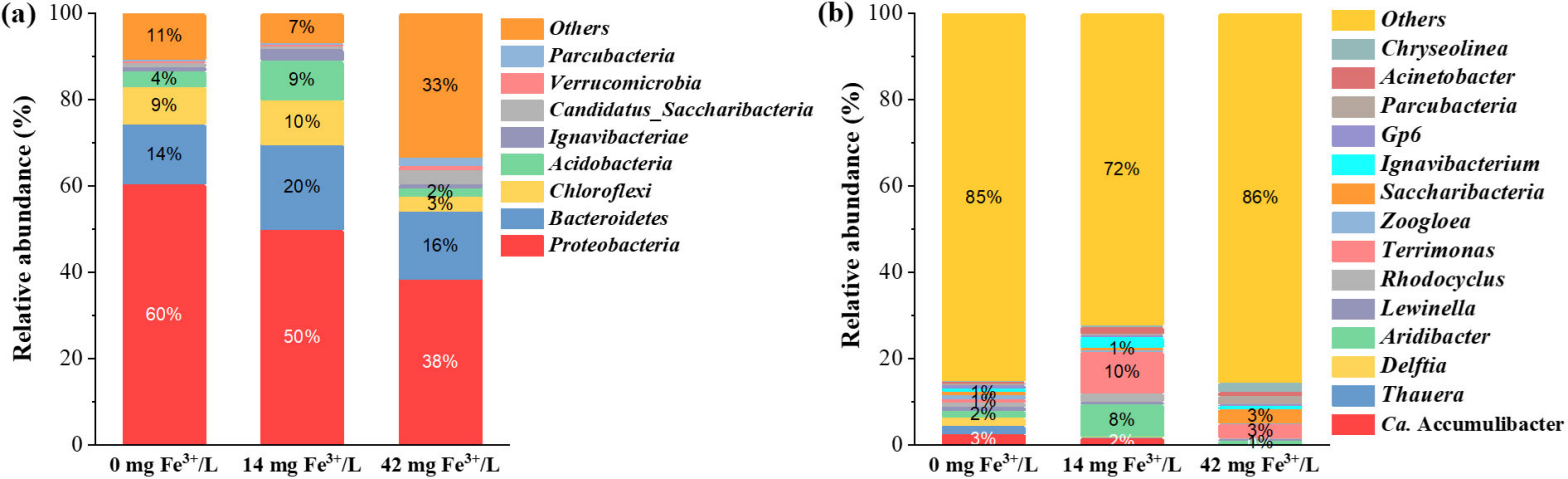
Relative abundance of microbial community throughout the operational periods at the phylum level **(a)** and at the genus level **(b)** (relative abundance > 0.5% in at least one sample).

The microbial community structure change was further analyzed to evaluate the potential influence of ferric salts. The top four relative abundances were Proteobacteria, Bacteroidetes, Chloroflexi, and Actinobacteria at the phylum level in all samples, as shown in Figure 5a. Proteobacteria were the most abundant phylum in all samples. The relative abundance of Proteobacteria was 60.37% (0 mg Fe^3+^/L), 49.91% (14 mg Fe^3+^/L), and 38.28% (42 mg Fe^3+^/L), respectively, which decreased with ferric addition. The microorganisms related to nitrogen removal and P removal predominantly belonged to Proteobacteria (Nguyen et al., 2011). The suppression of PAOs by ferric resulted in the drop of Proteobacteria abundance in the sludge sample after ferric addition.

As shown in Figure 5b, *Ca.* Accumulibacter was the most dominant genus in the sludge without ferric addition. *Ca.* Accumulibacter is a common PAOs (Li et al., 2024), and its relative abundance decreased significantly after adding ferric salts (one-way ANOVA, *p* < 0.05). The relative abundance of *Ca.* Accumulibacter decreased from 2.51% (0 mg Fe^3+^/L) to 0.21% (42 mg Fe^3+^/L). This might be attributed to the insufficient bioavailable P to PAOs. This corresponded to the deterioration of P release and uptake performance after adding ferric salts. The changes in microbial community structure demonstrated that the use of ferric salts had significant inhibitory effects on *Ca.* Accumulibacter, which would lead to the deterioration of biological P removal capability. In addition to *Ca.* Accumulibacter, *Aridibacter*, *Terrimonas*, and *Saccharibacteria* shifted as dominant bacteria after ferric addition. The genus *Aridibacter* and *Terrimonas* are aerobic bacteria (Huber et al., 2017; Jin et al., 2013). *Saccharibacteria* is capable of degrading various organic compounds under aerobic or anaerobic conditions (Kindaichi et al., 2016). These bacteria outcompeted PAOs to proliferate under high C/P conditions. The relative abundance of *Zoogloea* decreased with the addition of ferric salts, which dropped from 0.88% (0 mg Fe^3+^/L) to 0.13% (42 mg Fe^3+^/L). *Zoogloea* plays a central role in the formation of sludge floc by secreting EPS (An et al., 2016). This might explain the dramatic reduction of P in EPS after the addition of ferric salts.

## 4 Conclusion

Ferric salts had an inhibitory effect on the biological P removal process. Chemical P precipitation with ferric salts led to a significant rise of ortho-P in the sludge. This reduced the P available to PAOs and C/P ratio, and the metabolism of PAOs was inhibited under high C/P. The amount of P released and absorbed by PAOs dropped dramatically, resulting in a decrease of bioavailable P (NAIP, OP) and poly-P in sludge. The relative abundance of PAOs (*Ca.* Accumulibacter) decreased in the long-term operation. Due to the deteriorated biological P removal performance, the system relied more on chemical P removal.

## Data Availability

The data presented in the study are deposited in the Genome Sequence Archive repository (https://ngdc.cncb.ac.cn/gsa), accession number CRA031430.
